# Model Based Predictive Control of Multivariable Hammerstein Processes with Fuzzy Logic Hypercube Interpolated Models

**DOI:** 10.1371/journal.pone.0163116

**Published:** 2016-09-22

**Authors:** Daniel Cavalcanti Jeronymo, Antonio Augusto Rodrigues Coelho

**Affiliations:** 1 Computer Engineering Department, Federal University of Technology, Toledo, Paraná, Brazil; 2 Automation and Systems Engineering Graduate Program (PPGEAS), Federal University of Santa Catarina (UFSC), Florianópolis, Santa Catarina, Brazil; Chongqing University, CHINA

## Abstract

This paper introduces the Fuzzy Logic Hypercube Interpolator (FLHI) and demonstrates applications in control of multiple-input single-output (MISO) and multiple-input multiple-output (MIMO) processes with Hammerstein nonlinearities. FLHI consists of a Takagi-Sugeno fuzzy inference system where membership functions act as kernel functions of an interpolator. Conjunction of membership functions in an unitary hypercube space enables multivariable interpolation of N-dimensions. Membership functions act as interpolation kernels, such that choice of membership functions determines interpolation characteristics, allowing FLHI to behave as a nearest-neighbor, linear, cubic, spline or Lanczos interpolator, to name a few. The proposed interpolator is presented as a solution to the modeling problem of static nonlinearities since it is capable of modeling both a function and its inverse function. Three study cases from literature are presented, a single-input single-output (SISO) system, a MISO and a MIMO system. Good results are obtained regarding performance metrics such as set-point tracking, control variation and robustness. Results demonstrate applicability of the proposed method in modeling Hammerstein nonlinearities and their inverse functions for implementation of an output compensator with Model Based Predictive Control (MBPC), in particular Dynamic Matrix Control (DMC).

## Introduction

The origins of Model Based Predictive Control can be traced to Model Algorithmic Control (MAC) [1] and Dynamic Matrix Control [2–4]. In this type of controller a model is used to predict a process’ behavior, control actions are calculated aiming to minimize a cost function which is generally the quadratic error between a desired future setpoint and process’ output. MBPC is advantageous in relation to other control techniques such as PID (Proportional Integral Derivative) and LQR (Linear Quadratic Regulator) controllers since it can consider actuator constraints, process’ constraints and handle non-minimal and unstable processes as well as multivariable systems [5, 6].

**Fig 1:**
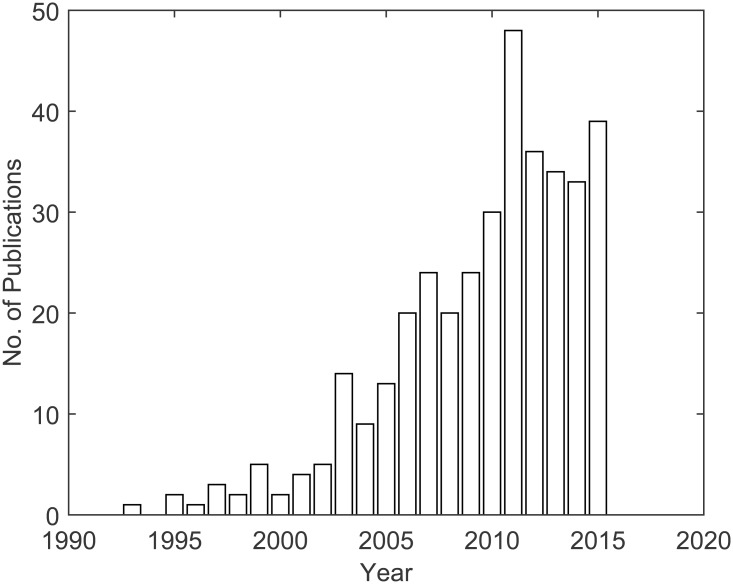
Approximate number of publications related to model predictive control with Hammerstein models. Source: Scopus’ database.

MBPC using Hammerstein models is still being investigated and applied both in numerical and experimental case studies, as depicted in Fig 1 (*Query* used in Scopus database: TITLE-ABS-KEY(“Model Predictive Control” hammerstein)), showing the number of related publications on journals and conferences over the last decades and demonstrating scientific and academic interest in the research and development of this area in process control.

Many processes, in particular distillation columns and chemical reactors, can be modeled by Hammerstein models [7], where a nonlinear and memoryless static function precedes linear dynamics. An appropriate model of the Hammerstein nonlinearity is necessary to achieve adequate process control. Different approaches can be found in literature regarding this problem. [8] employs an Artificial Neural Network (ANN) to model the inverse function of the Hammerstein nonlinearity in a self-tuning configuration, demonstrating good results regarding representation of Volterra series expansion of Hammerstein model. Similarly, [9–12] present results and methods regarding applications of ANNs in modeling and control of Hammerstein processes. [13] employs two independent DMC controllers, embedding nonlinear equations to deal with static nonlinearities. [14, 15] investigate the application of an output compensator based on the nonlinearity’s inverse function with DMC, in particular, [15] proposes a decision rule in the event of multiple solutions for the inverse nonlinearity. [16] follows similar approaches but uses fuzzy models identified by recursive least squares (RLS), the resulting fuzzy Hammerstein models are either a single-input single-output (SISO) or multiple-input single-output system, which can be analytically inverted to obtain the inverse nonlinearity. [17] models a solid oxide fuel cell’s nonlinearities using a multivariable fuzzy system and defines, similarly to [16], the inverse to be a straightforward analytical procedure since the resulting system is either SISO or MISO.

**Fig 2:**
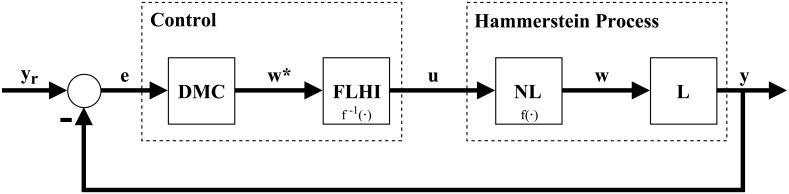
Control block diagram of proposal. DMC is designed taking into account linear dynamics from block L, FLHI is designed with data from nonlinear static gain block NL.

**Fig 3:**
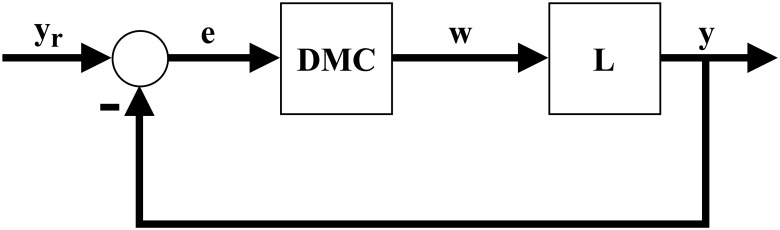
Resulting control block diagram of proposal. Cancellation between FLHI and NL originates a pseudo linear control problem.

In this paper we present an alternative solution to the control problem of systems with Hammerstein non-linearities. This solution can be applied to both monovariable or multivariable systems. Our solution is divided in two major steps. First, a general interpolator based on Takagi-Sugeno fuzzy logic is theorized and developed, named Fuzzy Logic Hypercube Interpolator, or FLHI, motivated by the necessity to adequately model static nonlinearities and its inverses. Second, FLHI is applied to modeling static nonlinearities and its inverses. These inverse models are employed as an output compensator for the predictive controller, resulting in an pseudo-linear system and allowing conventional linear control theory to be applied [8] to SISO, MISO or MIMO problems. Our proposal is as depicted in Figs 2 and 3, where *e* is the error signal between desired set-points *y*_*r*_ and process outputs *y*, w* is the control action from DMC, *u* is the control action modified by FLHI considering the nonlinear static gain from block NL and *w* is the output from the static nonlinearity. In ideal situations w* = *w*, however, modeling uncertainties account for differences between the two signals.

Similar approaches can be found in literature such as [16, 17], however, the following innovations are present and differentiate our work: i) fuzzy inference is defined on fuzzy logic operations, allowing changes such as choice of conjunction operator (t-norm) or choice of membership function to have major impacts on final results; ii) membership functions are used as kernel functions, allowing the interpolator to behave as nearest-neighbor, linear or cubic interpolator, to name a few; iii) the model’s point cloud input space is divided in convex regions and each region is projected (as in, mapped) to an unitary hypercube space, where interpolation occurs, this standardizes the input space from the fuzzy inference perspective and facilitates both interpolation and obtaining the inverse function; iv) inverse function is achieved by the solution of a nonlinear optimization problem for a known region, since the resulting fuzzy model is highly nonlinear depending on choices of conjunction operator or membership functions, as such, its inverse is not a “straightforward analytical procedure” as it is for other approaches in literature; v) multivalued (or multiset) inverse functions are adequately handled, as in multiple solutions are obtained if they exist, which allows for greater flexibility on control actions; vi) MIMO systems can be handled by our approach.

Results are presented for three study cases, a SISO system [18], a MISO system with uncoupled nonlinearities [19] and a MIMO system with highly coupled nonlinearities [20], presenting good results regarding control objectives such as reference tracking and minimization of control variation. Robustness considerations are also presented for cases where a mathematical model of the non-linearity is available.

The rest of this paper is organized as follows. DMC algorithm is presented for both monovariable and multivariable cases, further considerations are given in case of constraints as well as usage of output compensator for DMC. In what follows, foundations of the proposed Fuzzy Logic Hypercube Interpolator are presented. Then an overview and summary of the control problem using FLHI is given. Followed, are given considerations on robustness and performance metrics. Results are then presented for a SISO system, a MIMO system considering uncoupling and coupling of inputs and a MIMO system. In conclusion, final remarks are presented about the paper, the proposed method and future work.

## Methods

### Dynamic Matrix Control

Dynamic matrix control is one of the first model based predictive controllers, developed by Cutler and Ramaker [2–4]. Its internal model, the step response, is easily obtainable which allowed it to enjoy wide acceptance and industrial application, in particular chemical and oil [21] but also others such as automotive, food and aerospace [22]. Other advantages which contributed to its popularity are: applicability to multivariable systems; consideration of process constraints on inputs or outputs; prevention of excessive control actions; predictive reference tracking and disturbance rejection; to name a few [22, 23]. DMC’s Finite Step Response (FSR) internal model limits applications of the controler to open loop stable processes, however, alternatives are presented in literature [23, 24] for unstable processes.

In what follows the DMC algorithm is detailed according to [6, 23, 25], first for the SISO problem and then extended to the MIMO problem.

#### SISO DMC Design

DMC aims to reduce future tracking error and control action increments by minimization of the cost function:

$$J=\sum_{j=1}^{N_{y}}{\left[\hat{y}\left(t+j\right)-y_{r}\left(t+j\right)\right]}^{2}+\lambda \sum_{j=1}^{N_{u}}{\left[\Delta u(t+j-1)\right]}^{2}\;,$$ (1)

where $\hat{y}$ is the predicted process output *j* steps ahead given by a process model, *y*_*r*_ is the desired set-point, *N*_*y*_ is the prediction horizon, *N*_*u*_ is the control horizon and *λ* is the move suppression factor. Process output prediction is given by the finite step response model:

$$\hat{y}\left(t+j\right)=\sum_{i=1}^{j}g_{i}\Delta u\left(t+j-i\right)+f\left(t+j\right)\;,$$ (2)

*f* is the free response, dependent only on past variables:

$$f\left(t+j\right)=y\left(t\right)+\sum_{i=1}^{N}\left(g_{j+i}-g_{i}\right)\Delta u\left(t-i\right)\;.$$ (3)

Eqs (2) and (3) can be combined and rewritten in matrix form as:

$$\hat{y}=Gu+\underset{f}{\underset{︸}{Hu_{p}+y}}\;,$$ (4)

where:

$$\begin{array}{cc} {\hat{y}}^{T} & =\left[\hat{y}\left(t+1\right)\;\ldots \;\hat{y}\left(t+N_{y}\right)\right]\;,\\ u^{T} & =\left[\Delta u\left(t\right)\;\Delta u\left(t+1\right)\;\ldots \;\Delta u\left(t+N_{u}-1\right)\right]\;,\\ u_{p}^{T} & =\left[\Delta u\left(t-1\right)\;\Delta u\left(t-2\right)\;\ldots \;\Delta u\left(t-N_{u}\right)\right]\;,\\ y^{T} & =S^{T}y\left(t\right)\;. \end{array}$$ (5)

In Eqs (4) and (5) *S*^*T*^ is an unitary vector with dimensions *N*_*y*_ × 1, *G* is the dynamic matrix with dimension *N*_*y*_ × *N*_*u*_ and *H* is a matrix with dimension *N*_*y*_ × *N* − 1:

$$G=\left[\begin{array}{cccc} g_{1} & 0 & \cdots & 0\\ g_{2} & g_{1} & \cdots & 0\\ \vdots & \vdots & \ddots & \vdots \\ g_{N_{y}} & g_{N_{y}-1} & \cdots & g_{N_{y}-N_{u}+1} \end{array}\right]\;,$$ (6)

$$H=\left[\begin{array}{cccc} \left(g_{2}-g_{1}\right) & \left(g_{3}-g_{2}\right) & \cdots & \left(g_{N}-g_{N-1}\right)\\ \left(g_{3}-g_{1}\right) & \left(g_{4}-g_{2}\right) & \cdots & \left(g_{N+1}-g_{N-1}\right)\\ \vdots & \vdots & \ddots & \vdots \\ \left(g_{N_{y}+1}-g_{1}\right) & \left(g_{N_{y}+2}-g_{2}\right) & \cdots & \left(g_{N+N_{y}-1}-g_{N-1}\right) \end{array}\right]\;.$$ (7)

The functional Eq (1) can be rewritten in matrix form as:

$$J={\left(Gu+f-y_{r}\right)}^{T}\left(Gu+f-y_{r}\right)+\lambda \Delta u^{T}\Delta u\;,$$ (8)

optimization of the control law is given by the minimization of this quadratic cost function in terms of control action increments. This is achieved by differentiating *J* with respect to control action increments vector *u* and equating to zero, *i.e.* ∂*J*/∂Δ*u* = 0. The resulting control law is given by:

$$\Delta u=\underset{K_{dmc}}{\underset{︸}{{\left(G^{T}G+\lambda I\right)}^{-1}G^{T}}}\left(y_{r}-f\right)\;.$$ (9)

In practice, Eq (9) results in *N*_*u*_ control action increments, however, only Δ*u*(*t*) is used at each instant *t*. In the next instant *t* + 1 a new control action is calculated, this is known as sliding horizon control. Hence, only the first line of the gain matrix *K*_*dmc*_ is needed, which helps reduction of computational effort.

#### MIMO DMC Design

For MIMO processes the effect of each input variable to each output variable is described by its FSR. Eqs (1), (2) and (3) are affected and must be rewritten to account for these extra variables. This can be accomplished in matrix notation of Eq (4), which helps in obtaining a low verbosity solution.

Considering a system with *m* inputs and *n* outputs, Eq (5) is rewritten to:

$$\begin{array}{cc} {\hat{y}}^{T} & =\left[{\hat{y}}_{1}^{T}\;\ldots \;{\hat{y}}_{n}^{T}\right]\;,\\ u^{T} & =\left[u_{1}^{T}\;\ldots \;u_{m}^{T}\right]\;,\\ u_{p}^{T} & =\left[u_{p1}^{T}\;\ldots \;u_{pm}^{T}\right]\;,\\ y^{T} & =\left[y_{1}^{T}\;\ldots \;y_{n}^{T}\right]\;. \end{array}$$ (10)

Eqs (6) and (7) are rewritten in terms of *G*_*ij*_ and *H*_*ij*_, the SISO matrices, for the *i*-eth output and *j*-eth input, as:

$$G=\left[\begin{array}{cccc} G_{11} & G_{12} & \cdots & G_{1m}\\ G_{21} & G_{22} & \cdots & G_{1m}\\ \vdots & \vdots & \ddots & \vdots \\ G_{n1} & G_{n2} & \cdots & G_{nm} \end{array}\right]\;,$$ (11)

$$H=\left[\begin{array}{cccc} H_{11} & H_{12} & \cdots & H_{1m}\\ H_{21} & H_{22} & \cdots & H_{1m}\\ \vdots & \vdots & \ddots & \vdots \\ H_{n1} & H_{n2} & \cdots & H_{nm} \end{array}\right]\;,$$ (12)

Finally, the control law from Eq (9) can be applied considering a change of the vectors involved in prediction error:

$$y_{r}^{T}=\left[y_{r1}^{T}\;\ldots \;y_{rn}^{T}\right]\;,$$ (13)

$$f^{T}=\left[f_{1}^{T}\;\ldots \;f_{n}^{T}\right]\;.$$ (14)

#### Constraints

When constraints are considered the optimum solution is no longer the analytical solution of Eq (9). In this case, iterative methods for quadratic programming are necessary [26] and the control problem can be rewritten as:

$$\begin{array}{cccc} & \underset{\Delta u}{\text{minimize}} & & J=\frac{1}{2}\Delta u^{T}Q\Delta u+c^{T}\Delta u\\ & \text{subject}\;\text{to} & & A\Delta u\leq b \end{array}$$ (15)

where *Q* = *G*^*T*^ *G* + *λI*, *c* = *G*^*T*^(*f* − *y*_*r*_). *A* and *b* can be chosen to reflect limits on system variables such as, for example, control magnitude, process output magnitude or control increments [26].

### Fuzzy Logic Hypercube Interpolator

The main goal in control by output compensator approach is precise identification and modeling of a process’ non-linear characteristics so conventional linear control theory may be applied [8]. This problem motivated the creation of a general interpolator which exhibits desirable characteristics such as: modeling both a function and its inverse function; multivariable inputs and outputs; flexibility regarding interpolation characteristics; and, high computational efficiency.

In this section FLHI is presented according to it’s working algorithm, which is separated in three different parts. In the first part, a user provided point cloud is verified for consistency and defines an internal model which is used to feed posterior calculations. All pre-calculations occur in the first part, which acts as a setup for the interpolator. In the second part, function interpolation is defined by a Takagi-Sugeno fuzzy inference system in an unitary hypercube space. In the third part, inverse function interpolation is defined as a root finding problem in hypercube space in terms of an optimization problem.

#### Interpolant Setup

At this initial stage, the expected user input is a point cloud and the output are regions for interpolations and respective hypercubes, the main component of FLHI interpolant. The point cloud is a set $P=\left(xi_{i},xo_{i}\right){|}_{i=1}^{N}$, where *N* is the number of points in the point cloud, *xi* = (*xi*_1_, …, *xi*_*m*_) is a set of input coordinates of size *m* and *xo* = (*xo*_1_, …, *xo*_*n*_) is a set of output coordinates of size *n* such that the generating function of the point cloud is a mapping *f*: ℜ^*m*^ → ℜ^*n*^. In this context, hypercubes are interpolation regions where input coordinates *xi* are mapped to an unitary space in the range [0, 1]. The algorithm for interpolation is defined in Algorithm 1.

**Algorithm 1:** FLHI Interpolant Setup Algorithm

 **Input  :** a pointcloud *P*

 **Output  :** a set of *regions*

 // apply lexicographical ordering to the pointcloud according to input dimensions.

**1** *P* = lexicographical_order(*P*)

 // initialize as empty set.

**2** *regions* = ∅

**3** **for *each*** *base_point* ***in*** *P* **do**

  // if the point is at the border of the point cloud it can not generate a region, only belong in one.

**4**  **if** *is_border(base_point)* **then**

**5**   **continue**

**6**  **end**

  // get all (2⌃dimensions—1) points greater than the current point.

**7**  *neighbors* = find_greater_neighbors(*base_point*)

  // this region is formed by this point and its greater neighbors for a total of 2⌃dimensions points.

**8**  *region* = {*base_point*, *neighbors*}

  // region input coordinates are converted from problem coordinates to hypercube coordinates. output coordinates remain untouched. this is done by normalizing all points in all dimensions by its step size relative to the base point. *i.e.*, *base*_*point* has input coordinates (0, 0, …), the next point has coordinates (1, 0, …), the next (0, 1, …) and so on. this step turns the region into an hypercube, where input dimensions are bound between [0, 1].

**9**  *region*.*hypercube* = convert_region_to_hypercube_coordinates(*region*)

  // add this region to the output set

**10**  *regions*.add(*region*)

**11** **end**

A main characteristic of the point cloud is the distance between points for each dimension. A regular grid is determined by points which are equidistant across dimensions, that is in other words, with predetermined and uniform distances across dimensions. A semi-regular grid is determined by points with predetermined and non-uniform distances across dimensions. An irregular grid, *i.e.* scattered data, lacks structure or order regarding relative location of points.

Conversion from problem coordinates to unitary hypercube coordinates can be realized for a region by considering the base point as the null coordinate (0, 0, …) and adjusting each dimensions in all neighbor points in the region as either 1 if the neighbor’s dimension moves away from the base point or 0 if it remains unchanged. This can also be achieved by mapping all points in a region to hypercube coordinates by:

$$point\left(i\right).xi\left(j\right)=\frac{point(i).xi(j)-base\_point.xi(j)}{xi_{step(j)}}\;,$$ (16)

∀*i* ∈ (1, …, 2^*m*^), ∀*j* ∈ (1, …, *m*), where *m* is the amount of input dimensions and *xi*_*step*_ is the step size of a dimension, note *base*_*point* = *point*(1) in a region.

Current proposal focuses on a point cloud forming either a regular grid or semi-regular grid. Irregular grid, *i.e.* scattered data, remains as future work but some considerations are presented for such scenario in this paper. Two main challenges follow irregular data: i) tesselating necessary regions for interpolation; and ii) mapping an irregular region to a regular hypercube.

Tesselation of regions for surface reconstruction from scattered data is an open research topic [27] and further investigations are necessary to find or develop a suitable algorithm for application in FLHI. This is made further challenging by the fact current methods in literature focus on triangulations that require 3*^m^* points for a region, while FLHI is based on quadrangulations with requirement of 2*^m^* points.

Mapping of an irregular region to a regular hypercube is feasible by current algorithms applied in finite element methods, such as projective transform or bilinear transform [28] mappings.

#### Interpolation

FLHI Interpolation can be separated in three major procedures, summarized in Algorithm 2, Algorithm 3 and Algorithm 4.

In Algorithm 2, a search occurs to determine which region produced by FLHI setup Algorithm 1 delimits desired interpolation coordinates, in problem domain, *xi*. Once the region is determined, desired interpolation coordinates must be mapped to the hypercube established by the region.

**Algorithm 2:** FLHI Interpolate

 **Input  :** a set of *regions*, a set of input coordinates *xi*

 **Output  :** a set of output coordinates *xo*

 // find the region which contains the desired input coordinates.

**1** *region* = find_region_input_contains(*xi*)

 // convert the desired input coordinates to hypercube space.

**2** *xi* = convert_to_hypercube_coordinates(*xi*)

**3** *xo* = interpolate_hypercube(*region*.*hypercube*, *xi*)

With all coordinates now in hypercube domain, bounded by [0, 1], interpolation occurs in the hypercube as presented in Algorithm 2. The main concept of FLHI is that each of the 2*^m^* boundary points of the hypercube contain information about local geometry. Conjunction of information from all boundary points allows inference regarding true function value at any arbitrary position inside the hypercube. Thus, each of these points contribute with moment regarding whole hypercube area. Influence of each boundary point moment regarding any arbitrary position in the hypercube is inversely proportional to the distance between the boundary point and this arbitrary position.

**Fig 4:**
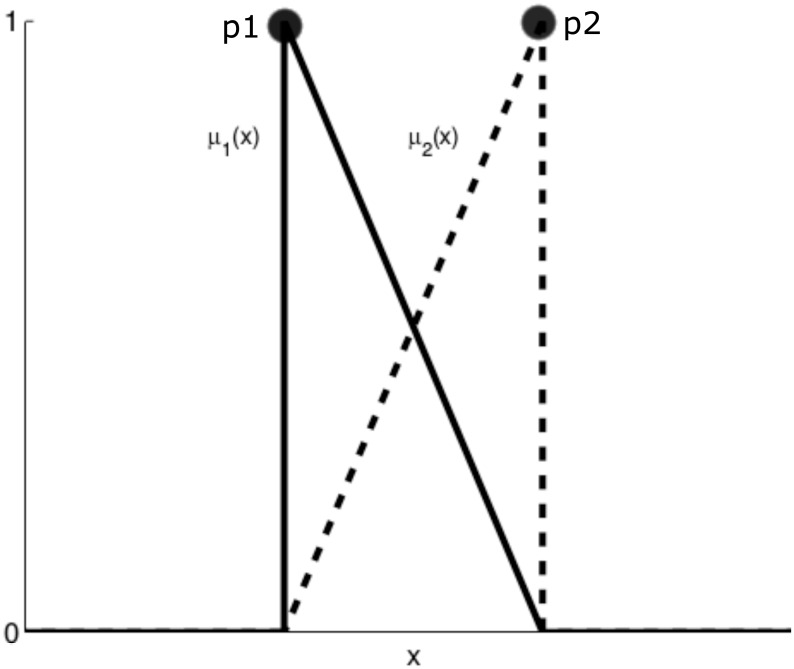
Graphical demonstration of influence of two points on the same dimension in unitary hypercube space.

Consider Fig 4, where two boundary points *p*1 and *p*2 are represented in the same input dimension *x* with unitary distance between each other. Each boundary point exhibits maximum, logical unitary, information at it’s own position, for it is a sampled function value, and this information’s contribution diminishes the further away from the point. Information contribution may be represented by a membership function that exhibits a maximum at the point’s location and diminishes the further away from the point. Furthermore, it is unnecessary to define two different membership functions on a single dimension for it can be determined one is the logical complement of the other, that is:

$$\mu_{2}\left(x\right)=1-\mu_{1}\left(x\right)\;.$$ (17)

Previous logic can be extended to any number of dimensions in a logical hypercube, such that a point has multiple local membership functions *lμ*, one for each dimension of which it is composed. Global membership for a boundary point can given by the logical conjunction of all local membership functions for that point. Thus, global membership *μ* of a boundary point is given by:

$$\mu \left(xi\right)=T\left(l\mu_{1},\ldots ,l\mu_{m}\right)\;,$$ (18)

where *T* is the triangular norm (t-norm). The applied norm can be any of Godel, Lukasiewicz, Hamacher, Product, etc. In this paper the applied t-norm for all cases was the product norm.

Finally, for each output dimension, an interpolated value can be obtained by first moment of area deffuzification:

$$FLHI\left(xi\right)=\sum_{i=1}^{2^{m}}\mu_{i}\left(xi\right)\cdot xo_{i}\;,$$ (19)

where *μ*_*i*_ and *xo*_*i*_ are, respectively, the global membership function and the output value of boundary point *i* in hypercube.

**Algorithm 3:** FLHI Interpolate Hypercube

 **Input  :** an *hypercube*, a set of input coordinates *xi*, count of input dimensions *m*, count of output dimensions *n*

 **Output  :** a set of output coordinates *xo*

 // initialize as empty set.

**1** *xo* = ∅

 // point count in region

**2** *point*_*count* = 2^*m*^

**3** **for** *xoIndex* = 1: *n* **do**

  // initialize a column vector of output values.

**4**  *xoVector* = zeros(*point*_*count*)

  // initialize a line vector for t-norm of membership functions.

**5**  *membershipVector* = zeros(*point*_*count*)

**6**  *point*_*index* = 0

**7**  **for *each*** *point* **in** *hypercube* **do**

   // empty set of membership functions for this point.

**8**   *membership* = ∅

   // evaluate the membership function for each dimension on this point.

**9**   **for** *xiIndex* = 1: *m* **do**

**10**    *mf* = evaluate_membership_function(*point*, *xi*, *xiIndex*)

**11**    *membership*.add(*mf*)

**12**  **end**

   // calculate t-norm for this point

**13**  *membershipVector*(*point*_*index*) = t_norm(*membership*)

   // set this point’s output value.

**14**  *xoVector*(*point*_*i*_*ndex*) = *point*.*xo*(*xoIndex*)

**15**  *point*_*index* = *point*_*index* + 1

**16** **end**

  // calculate interpolation value for this output by matrix product. this is achieved by moment of area deffuzification.

**17**  *xo*(*xoIndex*) = *membershipVector* ⋅ *xoVector*

**18** **end**

**Algorithm 4:** FLHI Evaluate Membership Function

 **Input  :** a *point* in hypercube space, a set of input coordinates in hypercube space *xi*, an index *xiIndex* of which input dimension is being calculated

 **Output  :** a membership function evaluation *mf*

**1** *x* = *xi*(*xiIndex*)

**2** *mf* = membership_function(*x*)

 // invert membership function logic if the point is situated on the right side of this dimension in hypercube (logical 1).

**3** **if** *point.xi(xiIndex) == 1* **then**

**4**  *mf* = 1 − *mf*

**5** **end**

Membership functions can be defined arbitrarily and different interpolators may be obtained by appropriate choice of membership function. In this paper the following membership functions are explored: nearest neighbor, linear, cubic, lanczos and spline.

#### Inverse Interpolation

Inverse interpolation in FLHI occurs as described in Algorithm 5 and begins by searching which regions may output the desired interpolation set *xo*. If the desired output set *xo* fits in maximum and minimum output coordinate boundaries for a region, by the intermediate value theorem this region may produce the desired output. This process can be computationally sped up if, in FLHI setup, maximum and minimum output coordinate boundaries are determined for each region as *a priori* knowledge.

It is important to note that choices of t-norm and membership functions that lead to well defined logical hypercube space, where global memberships are bound in [0, 1], limit this process to the evaluation of maximum and minimum values of *xo* for each boundary point. However, in ill-behaved logical hypercube spaces, particularly for parametric membership functions such as cubic, a search must be performed to determine maximum and minimum values for each region.

When a region is determined as being able to interpolate the desired output coordinate *xo*, a root search procedure is performed in terms of *xi*. This is defined as the minimization of sum of squared residual errors between interpolation in this region and the expected output coordinates:

$$\begin{array}{cccc} & \underset{xi}{\text{minimize}} & & \sum_{j=1}^{n}{\left(xo_{j}-FLHI\left(xi\right)\right)}^{2} \end{array}$$ (20)

**Algorithm 5:** FLHI Interpolate Inverse

 **Input  :** a set of *regions*, a set of desired output coordinates *xo*

 **Output  :** a set of input coordinates *xi*

 // initialize as empty set

**1** *xi* = ∅

 // check each region to see if its maximum and minimum outputs contain *xo* when continuous t-norm and membership functions are applied, interpolated space is continuous and existance of *xo* is guaranteed by the intermediate value theorem.

**2** **for *each*** *region* ***in*** *regions* **do**

**3**  **if** *region_output_contains(region, xo)* **then**

   // solve a minimization problem on variable *xi* using objective function, the sum of squared residuals.

**4**   x = minimize(objective_function, *region*.*hypercube*, *xo*)

   // convert hypercube coordinates to original problem coordinates.

**5**   *x* = convert_to_problem_coordinates(*x*)

   // add to set of solutions.

**6**   *xi*.add(*x*)

**7**  **end**

**8** **end**

Multiple regions may contain the desired output coordinates. As such, inverse interpolation is a multivalued function and may return multiple sets of solutions.

### Control Algorithm Summary

This is the set of steps which summarize the proposed control approach in practice:

- Setup a DMC controller with desired parameters *N*_*y*_, *N*_*u*_, *N*, *λ* and a step model from the linear block of the Hammerstein model;
- Setup a FLHI interpolant with nonlinearity data from the static nonlinearity block of the Hammerstein model;
- At each control instant, obtain process output and calculate the necessary linear control action of *w*(*t*) considering constraints on upper and lower bounds of nonlinearity;
- Apply FLHI inverse interpolation with desired membership function on *w*(*t*) to produce the desired control signal *u*(*t*);
- In case of multiple solutions from inverse interpolation, choose the one which minimizes control variation Δ*u*(*t*);
- Control loop is repeated as necessary.

**Algorithm 6:** FLHI Objective Function

 **Input  :** a set of test input coordinates *xi*, a set of expected output coordinates *exo*, an *hypercube*

 **Output  :** sum of squared residuals *error* between *exo* and the evaluation of FLHI at *xi*

 // interpolate hypercube in desired position.

**1** *xo* = interpolate_hypercube(*hypercube*, *xi*)

 // output sum of squared residuals.

**2** error = sum((*xo* − *exo*)^2^)

### Considerations on robustness of output compensation—Multiplicative gain uncertainty

**Fig 5:**
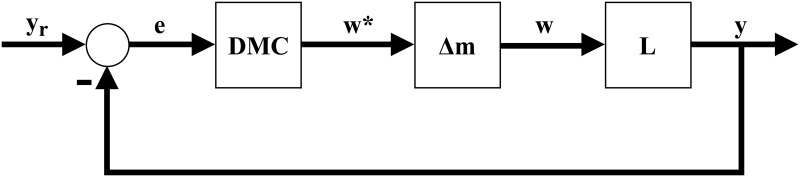
Input gain model uncertainties in cancellation between FLHI and NL.

Cancellation between the static nonlinearity function *f* and inverse model *f*^−1^ of FLHI is given by:

$$w\left(t\right)=f(f^{-1}\left(w^{*}\left(t\right)\right))\;,$$ (21)

such that in ideal conditions *w*(*t*) = w*(*t*). However, in practice, accuracy of FLHI’s inverse model is not perfect but rather an approximation of missing information from the point cloud. This model uncertainty can be represented in Fig 3 by re-arranging the blocks as in Fig 5, where Δ_*m*_ is an input gain uncertainty:

$$\Delta_{m}=\frac{w(t)}{w^{*}\left(t\right)}=\frac{f(f^{-1}\left(w^{*}\left(t\right)\right))}{w^{*}\left(t\right)}\;,$$ (22)

where ideally the input gain uncertainty Δ_*m*_ = 1.

#### Output Compensation Stability Theorem

A system controlled by output compensation is asymptotically stable if the following necessary and sufficient condition is met:

$$\begin{array}{cc} \left|\right|\Delta_{m}{\left|\right|}_{\infty } & <GM\;,\\ PM & >0{}^{\circ}\;, \end{array}$$ (23)

where *GM* and *PM* are respectively the gain margin and phase margin of the open loop gain *H* = *DMC* ⋅ *L*, and |Δ_*m*_|_∞_ is the H-infinity norm of the input gain model uncertainty.

*Proof*. A feedback, closed loop, system is asymptotically stable if the Bode-Nyquist stability criterion is met:

$$\begin{array}{cc} GM & >1\;,\\ PM & >0{}^{\circ}\;. \end{array}$$ (24)

Considering *GM* = 1/|*H*(*jw*_*pc*_)|, where *jw*_*pc*_ is the phase crossing frequency and *H* is the open loop gain of an arbitrary system, Eq (24) becomes:

$$\begin{array}{cc} \frac{1}{\left|H(jw_{pc})\right|} & >1\;,\\ PM & >0{}^{\circ}\;. \end{array}$$ (25)

Now, substituting the open loop gain of Fig 5 which is given by *H* = *DMC* ⋅ Δ_*m*_ ⋅ *L*:

$$\begin{array}{cc} \frac{1}{|DMC\left(jw_{pc}\right)\cdot \Delta_{m}\left(jw_{pc}\right)\cdot L\left(jw_{pc}\right)|} & >1\;,\\ PM & >0{}^{\circ}\;. \end{array}$$ (26)

Considering the input gain uncertainty Δ_*m*_ is itself a static nonlinear gain that does not depend on frequency response, its worst case is given by its H-infinity norm ||Δ_*m*_||_∞_ and *H* = *DMC* ⋅ *L*:

$$\begin{array}{cc} \frac{1}{\left|\right|\Delta_{m}{\left|\right|}_{\infty }}\cdot \underset{GM}{\underset{︸}{\frac{1}{\left|H(jw_{pc})\right|}}} & >1\;,\\ PM & >0{}^{\circ}\;. \end{array}$$ (27)

Finally, by arranging terms Eq (23) is obtained:

$$\begin{array}{cc} \left|\right|\Delta_{m}{\left|\right|}_{\infty } & <GM\;,\\ PM & >0{}^{\circ}\;. \end{array}$$ (28)

#### Measuring worst case model error

A definition of stability with output compensation control is given by Eq (23) considering worst case model error ||Δ_*m*_||_∞_ as a robustness metric in relation to gain margin. Model absolute relative error (MARE) can be represented by:

$$MARE\left(x_{i}\right)=\left|\frac{NL(FLHI\left(x_{i}\right))}{x_{i}}\right|\;,$$ (29)

then, worst case model error becomes a maximization problem:

$$\begin{array}{cccc} \left|\right|\Delta_{m}{\left|\right|}_{\infty }=\; & \underset{x_{i}}{\text{maximize}} & & MARE(x_{i})\\ & \text{subject}\;\text{to} & & x_{i}\leq ub\\ & & & x_{i}\geq lb\;, \end{array}$$ (30)

where *ub* and *lb* are respectively upper and lower bounds of input space *x*_*i*_.

In practice the true nonlinearity *NL* is unknown but it is either mathematically or computationally modeled. In cases where only a point cloud from a real data set is available, this approach can be useful to measure the trade-off between a simple and a more complex model.

Given the locality nature of FLHI, originated from regions of interpolations, local optimization techniques are neither capable or satisfactory in solving Eq (30). Global search methods are necessary such as [29].

### Performance metrics

In this section a performance metric is proposed to evaluate the effectiveness of different membership functions in FLHI models and its effects on set point tracking and control action. A fair assessment can be realized when the performance metric mimics the cost function Eq (1) of the model based predictive controller.

Set point tracking is evaluated by Integral Squared Error (ISE) of all outputs and output references:

$$ISE=\sum_{i=1}^{n}{\left(y_{i}-y_{r_{i}}\right)}^{2}\;.$$ (31)

Control efforts are measured by the Integral Squared Variation of Control (ISVC):

$$ISVC=\sum_{i=1}^{m}\Delta u_{i}^{2}\;.$$ (32)

The last performance metric aims to mimic DMC’s cost function Eq (1) and its purpose is to provide overall assessment of results:

J=ISE+λ·ISVC. (33)

A final remark of caution is presented in regards to analysis of results. All results include an ideal case where only the linear process is controlled, disregarding nonlinearities. This ideal linear case is included to provide an estimate of optimal set point tracking and control variation, however, *ISVC* and *J* metrics for ideal cases consider the linear control signal *w*(*t*) instead of nonlinear control signal *u*(*t*), which is inexistent in these scenarios. Therefore disparities can be observed regarding *ISVC* and *J* metrics of ideal cases in contrast to nonlinear cases since different control magnitudes are involved, due to effects of static nonlinearities.

## Results

In this section results are presented for three case studies in order to demonstrate the proposed method. The first study case regards a SISO system described in [18, 30] where the nonlinearity is a fourth order polynomial. The second is a MISO system described in [19], its input nonlinearities are described by third order uncoupled polynomials. The third is a MIMO system described in [20, 31], this system exhibits highly coupled input nonlinearities with exponential and polynomial terms.

For all case studies FLHI is used to model the system’s nonlinear portion and its inverse for application in output compensation, then, DMC is designed considering the model’s linear dynamics. Control action and process output dynamics are presented for all study cases as well as comparisons on the effects of different membership functions on DMC’s cost function of Eq (1) and its relating performance indices ISE and ISVC. Results include the ideal scenario, where nonlinearities are ignored and only the linear system is controlled, as well as different membership functions such as nearest neighbor, linear, cubic, lanczos and spline.

### SISO Study Case

A distillation column is modeled as a Hammerstein system and presented in [18]. In the original work [18], two models are given, one with a third order polynomial for the input nonlinearity and another with a fourth order polynomial. Both models exhibit a first order linearity.

A typical application of output compensation control [8] in this scenario would focus on the third order polynomial model since this can be trivially inverted and guarantees at least one real solution, being of uneven order. Despite being more accurate, the fourth order polynomial presents a problem in practical applications since its analytical inversion could lead to imaginary roots and an absence of feasible solution.

Our proposed method in this paper does not suffer from the problem of imaginary roots since model inversion occurs in the problem’s universe of discourse. As such, parity of model order is not a problem for our approach. From [18, 30], the fourth order polynomial Hammerstein model is:

$$\begin{array}{cc} w(t) & =1.04u\left(t\right)-14.11u{\left(t\right)}^{2}-16.72u{\left(t\right)}^{3}+562.75u{\left(t\right)}^{4}\;,\\ G(z^{-1}) & =\frac{0.243z^{-1}}{1-0.757z^{-1}}\;. \end{array}$$ (34)

**Fig 6:**
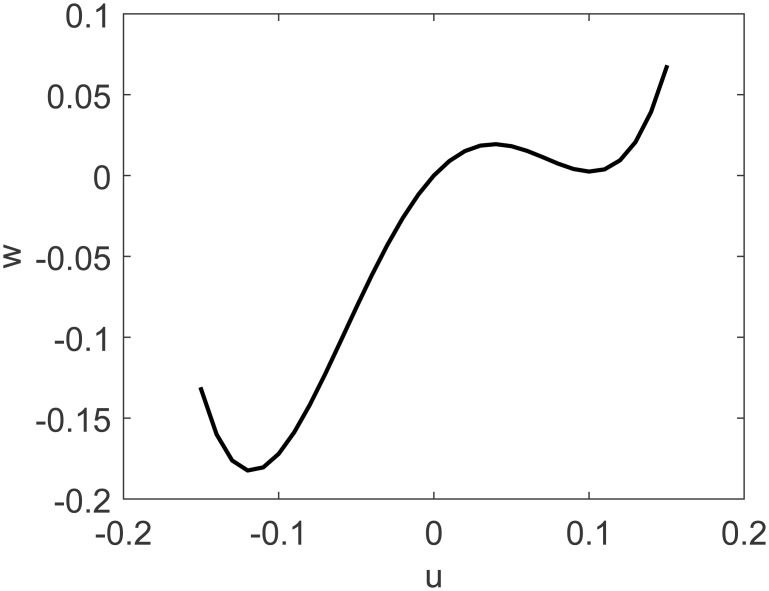
Fourth order polynomial nonlinearity of SISO study case.

A representation of the nonlinearity in Eq (34) is presented in Fig 6. DMC parameters for this problem are: model horizon *N* = 26, prediction horizon *N*_*y*_ = 5, control horizon *N*_*u*_ = 1 and control movement suppression factor *λ* = 1. Desired set point *y*_*r*_ is moved between 0.01, −0.05 and 0.06. Control signal *u* is limited in the range [−0.15, 0.15].

**Fig 7:**
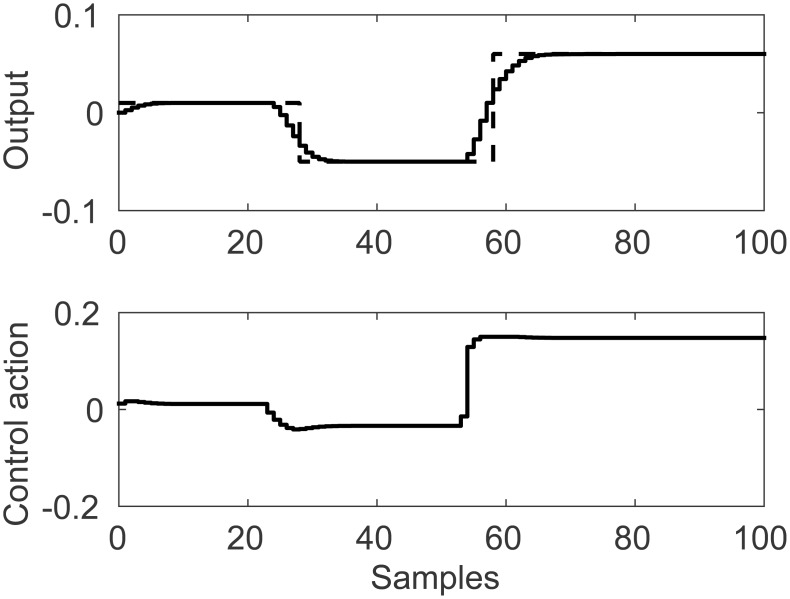
Simulation results for SISO control study case with linear membership function.

Control results for FLHI with linear membership functions are presented in Fig 7. A comparison of the effects of different membership functions considering DMC’s cost function is given in Table 1. For this study case, FLHI with nearest neighbor membership function fails to achieve reference tracking for all set-points and exhibits control ringing on the first set-point.

**Table 1:** Comparison of results for SISO control study case considering performance indices.

Membership Function	ISE	ISVC	J
Ideal	0.01089	0.0215	0.03239
Nearest Neighbor	0.01622	0.0253	0.04152
Linear	0.01085	0.02197	0.03282
Cubic	0.01086	0.02208	0.03294
Lanczos	0.01112	0.02255	0.03368
Spline	0.01089	0.01533	0.02621

Robustness considerations are given for this study case in order to compare the effects of different membership functions on robustness. Robustness indices for this study case are *GM* = 10.3221, *PM* = 72.253 and maximum sensitivity *M*_*s*_ = 1.226. Worst case model error considering MARE metric for each membership function is presented in Table 2, demonstrating nearest neighbor as the worst model and linear as the best. Stability criteria of Eq (23) is well met for all membership functions.

**Table 2:** Comparison of results for SISO control study case considering worst case model error metric.

Membership Function	||Δ_*m*_||_∞_
Nearest Neighbor	3.2201
Linear	2.9410
Cubic	2.9405
Lanczos	2.9622
Spline	2.9901

### MISO Study Case

A Hammerstein system is proposed and employed in [19] for benchmarking a model identification method. This system, given in Eq (35), presents two inputs with uncoupled third order polynomial nonlinearities. The linear subsystem presents coupling between inputs [32, 33] according to its Relative Gain Array (RGA) in Eq (36).

$$\begin{array}{cc} w1(t) & =u_{1}\left(t\right)+4u_{1}^{2}\left(t\right)+1.5u_{1}^{3}\left(t\right)\;,\\ y_{1}\left(t\right) & =\frac{0.1333z^{-1}+0.0667z^{-2}}{1-1.5z^{-1}+0.7z^{-2}}w_{1}\left(t\right)\;,\\ w2(t) & =u_{2}\left(t\right)+3u_{2}^{2}\left(t\right)+2u_{2}^{3}\left(t\right)\;,\\ y_{2}\left(t\right) & =\frac{0.4z^{-1}+0.3z^{-2}}{1-0.9z^{-1}+0.6z^{-2}}w_{2}\left(t\right)\;,\\ y(t) & =y_{1}\left(t\right)+y_{2}\left(t\right); \end{array}$$ (35)

$$RGA=\left[\begin{array}{cc} 0.5 & 0.5 \end{array}\right]$$ (36)

DMC parameters for this problem are: model horizon *N* = 31, prediction horizon *N*_*y*_ = 10, control horizon *N*_*u*_ = 5 and control movement suppression factor *λ* = 1. Desired set point *y*_*r*_ is moved between 10, 50 and −5. Control signal *u* is bound in the range [−2, 2].

This case study is presented in two parts plus remarks. First, nonlinearities are modeled separately by FLHI considering the known nonlinear uncoupling. Secondly, nonlinearities are modeled in combination by FLHI as if its nonlinear uncoupling was unknown. The expected outcome is of identical results for both scenarios, however, with a considerably higher computational load on the second scenario, both for FLHI model creation and model inversion.

#### Uncoupled model and control

**Fig 8:**
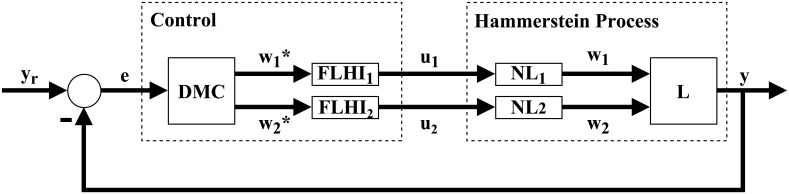
Block diagram of MISO case study considering knowledge of uncoupling in nonlinearities. Two reduced FLHI models are used, one for each nonlinearity.

When knowledge of uncoupling of nonlinearities is available it can greatly reduce computational needs. As such, in this first instance, this problem is modeled considering this knowledge. Control block diagram of this first approach is presented in Fig 8.

**Fig 9:**
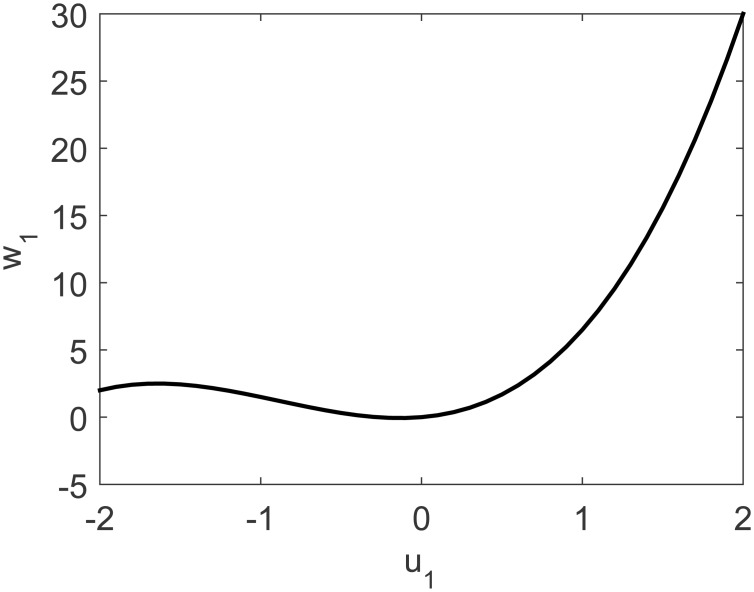
Uncoupled nonlinearity of MISO study case for first input.

**Fig 10:**
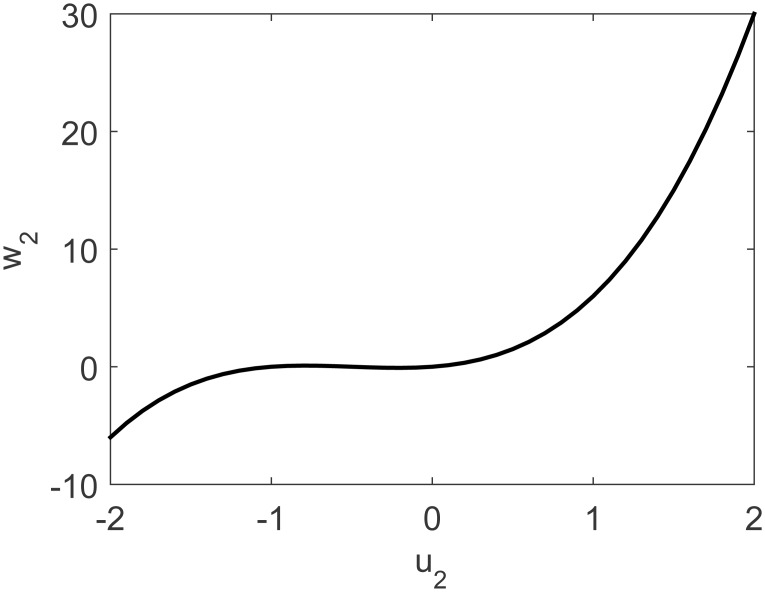
Uncoupled nonlinearity of MISO study case for second input.

**Fig 11:**
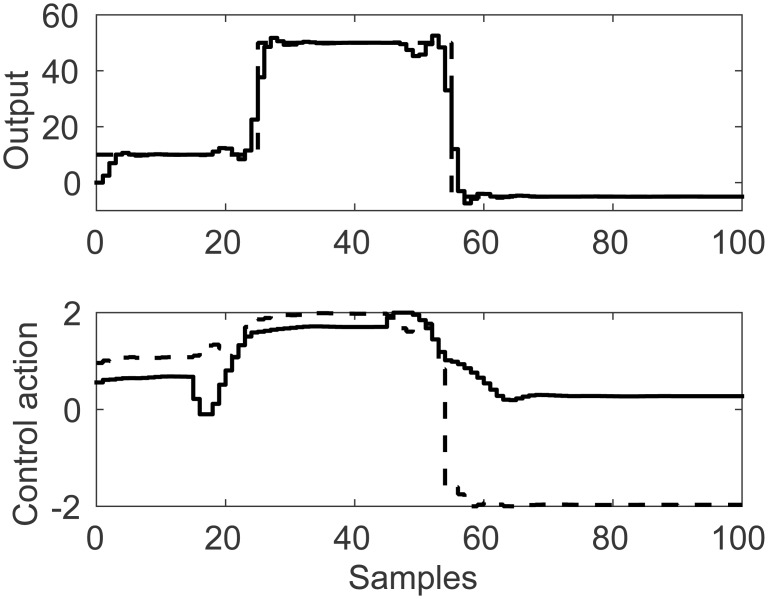
Simulation results for MISO uncoupled control study case with linear membership function. Light continuous line represents the second input, dark continuous lines represent first input and output.

A representation of the nonlinearity in Eq (35) is presented in Figs 9 and 10. Process output and control actions are depicted in Fig 11. Performance indices and cost function are presented in Table 3. Regarding computational load, FLHI required a point cloud of 41 points and unitary hypercube dimension.

**Table 3:** Comparison of results for MISO uncoupled control study case considering performance indices.

Membership Function	ISE	ISVC	J
Ideal	1145	8.34	1153
Nearest Neighbor	1204	8.41	1212
Linear	1146	8.113	1154
Cubic	1143	8.139	1151
Lanczos	1162	7.967	1170
Spline	1143	8.245	1151

#### Coupled model and control

**Fig 12:**
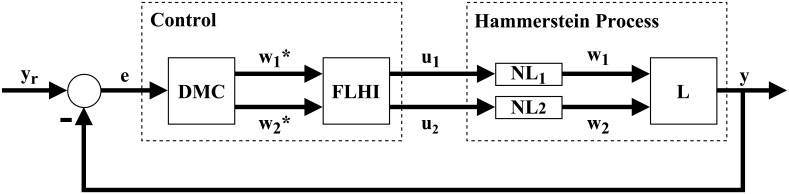
Block diagram of MISO case study considering coupling in nonlinearities. A full FLHI model is used considering both nonlinearities.

When coupling of nonlinearities is present or unknown, FLHI can be employed to model multivariable relationships. In this second instance, FLHI is applied considering both input variables are coupled even though in practice they are uncoupled. Control block diagram of this approach is presented in Fig 12.

**Fig 13:**
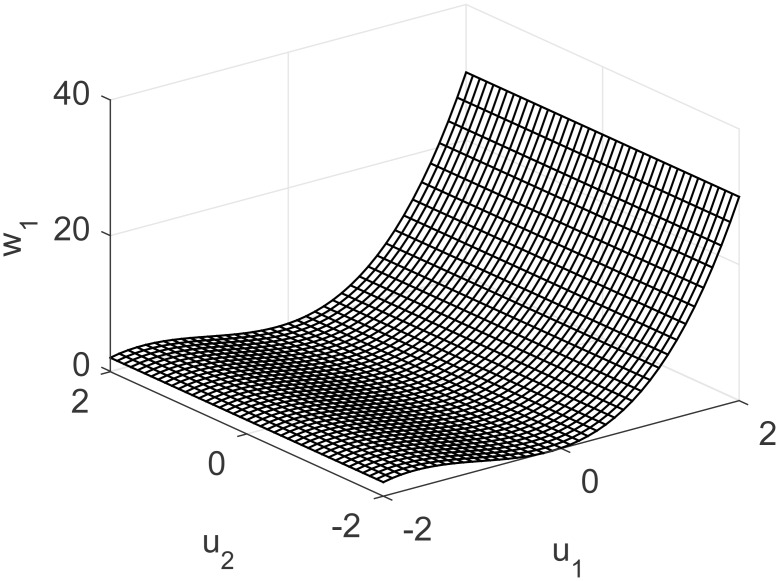
Nonlinearity of MISO study case for first output considering unknown coupling.

**Fig 14:**
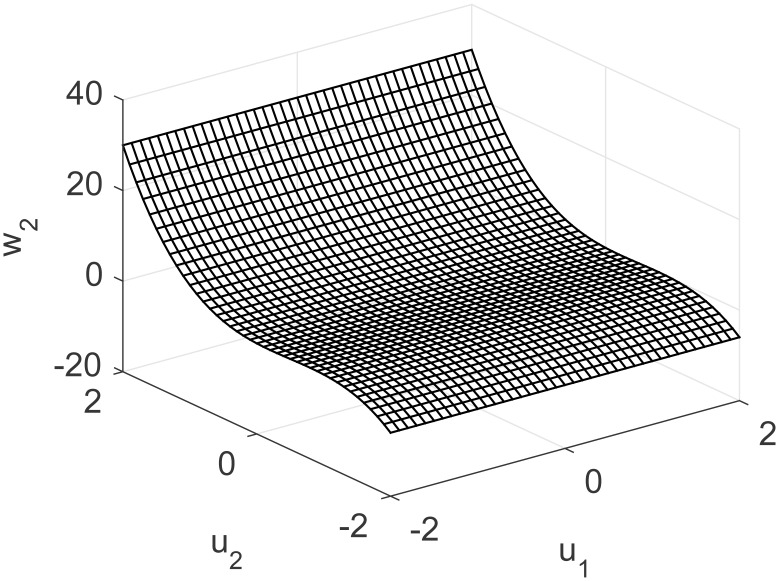
Nonlinearity of MISO study case for second output considering unknown coupling.

**Fig 15:**
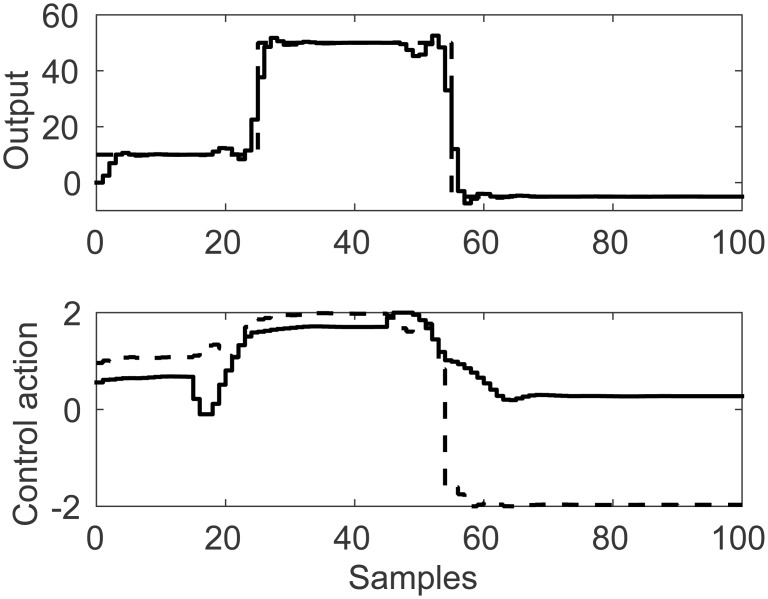
Simulation results for MISO coupled control study case with linear membership function. Light continuous line represents the second input, dark continuous lines represent first input and output.

A representation of the nonlinearity in Eq (35) is presented in Figs 13 and 14, considering coupling and a nonlinear multivariable model. Process output and control actions are depicted in Fig 15. Performance indices and cost function are presented in Table 4. Regarding computational load, FLHI required a point cloud of 1681 points and a two dimensional hypercube, respective to the system’s input dimensions.

**Table 4:** Comparison of results for MISO uncoupled control study case considering performance indices.

Membership Function	ISE	ISVC	J
Ideal	1145	8.34	1153
Nearest Neighbor	1204	8.41	1212
Linear	1146	8.113	1154
Cubic	1143	8.139	1151
Lanczos	1162	7.967	1170
Spline	1143	8.245	1151

#### MISO Remarks

Set point was tracked in all cases and no ringing or abnormal control actions were observed. High ISE and J indices are explained by the very large jumps between set points.

Results were as expected regarding identical process response and performance indices for both uncoupled and coupled scenarios. A more complex model in this study case does not bring any benefit since the same behavior of uncoupling is modeled in both instances. FLHI’s increase in computational load in the second scenario is expected since the necessarily larger point cloud is a combination of both inputs and the internal hypercube mimics input dimensions, adding to the model’s complexity.

### MIMO Study Case

A MIMO Hammerstein model is proposed in [20] as a benchmark for model identification. This process exhibits two inputs and two outputs, where inputs present heavily coupled nonlinearities and lightly coupled linearities. This Hammerstein model is described as follows:

$$\begin{array}{cc} \left[\begin{array}{c} w_{1}\left(t\right)\\ w_{2}\left(t\right) \end{array}\right] & =\left[\begin{array}{c} u_{1}{\left(t\right)}^{3}-u_{1}\left(t\right)u_{2}\left(t\right)+2u_{2}{\left(t\right)}^{2}\\ 0.582(e^{\left(u_{1}\left(t\right)+u_{2}\left(t\right)\right)}-1) \end{array}\right]\;,\\ \left[\begin{array}{c} y_{1}\left(t\right)\\ y_{2}\left(t\right) \end{array}\right] & =\left[\begin{array}{cc} \frac{0.1z^{-1}+0.2z^{-2}}{1-1.2z^{-1}+0.35z^{-2}} & \frac{z^{-1}}{1-0.7z^{-1}}\\ \frac{0.3z^{-1}+0.2z^{-2}}{1-0.8z^{-1}} & \frac{z^{-1}+0.5z^{-2}}{1+0.4z^{-1}} \end{array}\right]\left[\begin{array}{c} w_{1}\left(t\right)\\ w_{2}\left(t\right) \end{array}\right]\;. \end{array}$$ (37)

**Fig 16:**
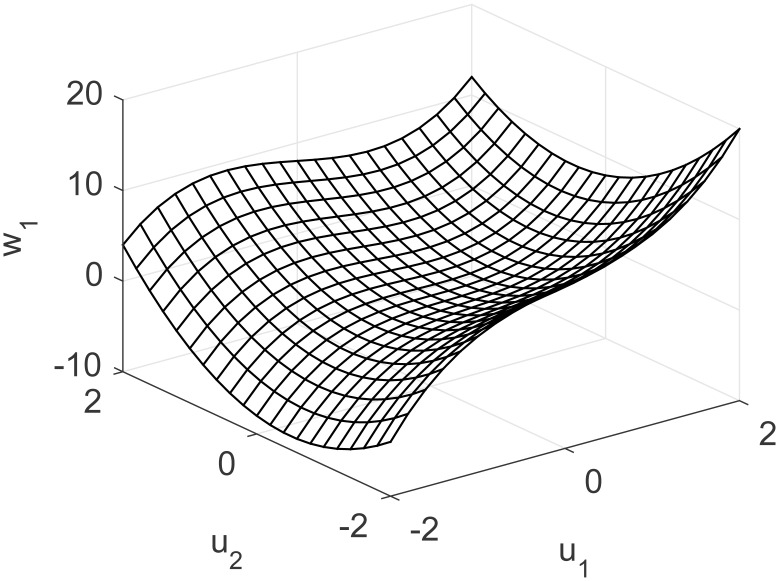
Nonlinearity of MIMO study case for first output.

**Fig 17:**
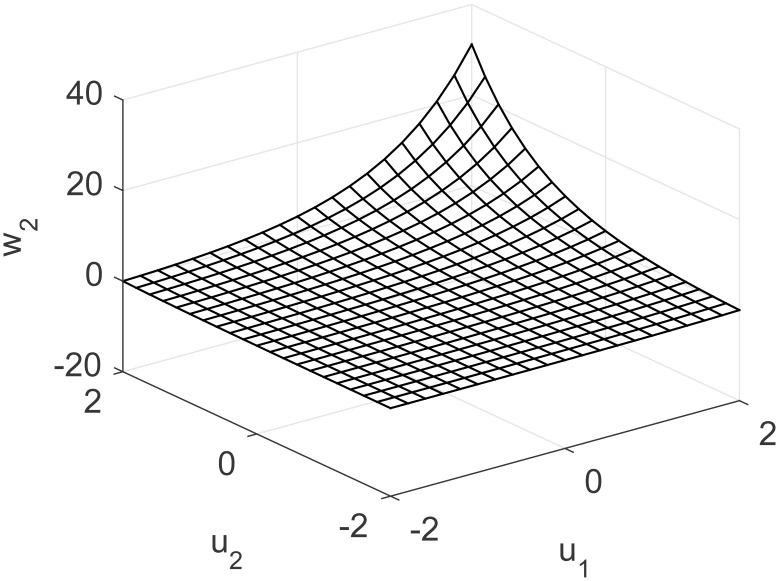
Nonlinearity of MIMO study case for second output.

A representation of the nonlinearity in Eq (37) is presented in Figs 16 and 17, exhibiting strong coupling and a nonlinear multivariable model. DMC parameters for this problem are: model horizon *N* = 31, prediction horizon *N*_*y*_ = 15, control horizon *N*_*u*_ = 5 and control movement suppression factor *λ* = 4. Desired set point for the first output *y*_*r*1_ is moved between 11 and 15, second set point *y*_*r*2_ is moved between 6, 10, 13 and 10. Control signal *u* is bound in the range [−2, 2].

**Fig 18:**
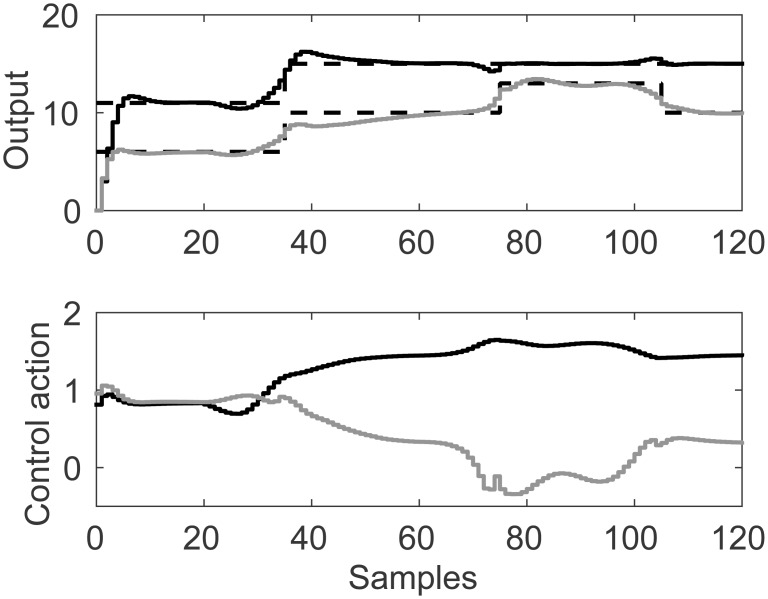
Simulation results for MIMO control study case with linear membership function. Light continuous line represents second input and output, dark continuous lines represent first input and output.

Simulation results for linear membership function are presented in Fig 18 and performance indices in Table 5. Nearest neighbor results present noisy set point tracking and heavy control ringing. Other results present very light oscillations in control on the second set point change for *y*_*r*2_. Coupling effects can be noticed in later set point changes.

**Table 5:** Comparison of results for MIMO uncoupled control study case considering performance indices.

Membership Function	ISE	ISVC	J
Ideal	261.1	0.52	263.7
Nearest Neighbor	363.6	2.88	378
Linear	322.4	0.2922	323.9
Cubic	320.3	0.2929	321.8
Lanczos	334.8	0.4897	337.2
Spline	324.2	0.3603	326

## Discussion

In this paper a novel method for modeling nonlinearities has been presented and applied to the problem of Hammerstein control using output compensation. The fuzzy logic hypercube interpolator, or FLHI for short, builds a fuzzy model based on point cloud data and allows model inversion. Model inversion enables FLHI to be applied directly as an output compensator, transforming the nonlinear control problem in a pseudo linear problem. Output compensation control, like in [8, 16], is not related or anywhere similar to linearizing control [34, 35].

Results are presented for a SISO process, a MISO process with uncoupled and coupled cases, and a MIMO process. These results include the ideal scenario, where only the linear system is controlled, and practical scenarios where the nonlinear Hammerstein system is controlled. In practical cases FLHI is applied using different membership functions such as Nearest Neighbor, Linear, Cubic, Lanczos and Spline. These results indicate the applicability of FLHI in both modeling of Hammerstein nonlinearities and output compensation, from its model inversion.

FLHI is currently limited to regular or semi-regular grid point clouds and injective data. Multivalued, *i.e.* non-injective, data and irregular grids are not automatically supported by the current method. Multivalued data can be used with FLHI but it is necessary for it to be manually separated in injective sets. Extrapolation is currently unsupported but the method can be trivially extended for it.

Future work includes, but is not limited to: i) support for irregular grids, as a possibility based on kd-trees; ii) support for multivalued data, using branch cuts; iii) study and analysis of multiplicative uncertainties, modeling errors, created by FLHI in its application as an output compensator; iv) investigation of FLHI in modeling unknown Hammerstein nonlinearities; v) investigation of the applicability of FLHI in other control situations where model inversion is necessary; vi) study of different membership functions, in particular parametric ones similar to cubic; vii) study of different fuzzy t-norms, logical conjunction between membership functions.
